# Patterns of cortical grey matter thickness reduction in multiple sclerosis

**DOI:** 10.1002/brb3.2050

**Published:** 2021-01-27

**Authors:** Juichi Fujimori, Kazuo Fujihara, Mike Wattjes, Ichiro Nakashima

**Affiliations:** ^1^ Division of Neurology Tohoku Medical and Pharmaceutical University Sendai Japan; ^2^ Department of Multiple Sclerosis Therapeutics Tohoku University Graduate School of Medicine Sendai Japan; ^3^ Department of Multiple Sclerosis Therapeutics Fukushima Medical University School of Medicine and Multiple Sclerosis and Neuromyelitis Optica Center Southern Tohoku Research Institute for Neuroscience Koriyama Japan; ^4^ Department of Diagnostic and Interventional Neuroradiology Hannover Medical School Hannover Germany

**Keywords:** cluster analysis, cortical gray matter, MRI, multiple sclerosis, temporal pole

## Abstract

**Objective:**

To examine the patterns of cortical gray matter thickness in multiple sclerosis (MS) patients.

**Methods:**

Seventy‐four MS patients—clinically isolated syndrome (4%), relapsing–remitting MS (79%), and progressive MS (17%)—and 21 healthy controls (HCs) underwent 1.5 Tesla T1‐weighted 3D MRI examinations to measure brain cortical thickness in a total of 68 regions of interest. Using hierarchical cluster analysis with multivariate cortical thickness data, cortical thickness reduction patterns were cross‐sectionally investigated in MS patients.

**Results:**

The MS patients were grouped into three major clusters (Clusters 1, 2, and 3). Most of the regional cortical thickness values were equivalent between the HCs and Cluster 1, but decreased in the order of Clusters 2 and 3. Only the thicknesses of the temporal lobe cortices (the bilateral superior and left middle temporal cortex, as well as the left fusiform cortex) were significantly different among Clusters 1, 2, and 3. In contrast, temporal pole thickness reduction was evident exclusively in Cluster 3, which was also characterized by increased lesion loads in the temporal pole and the adjacent juxtacortical white matter, dilatation of the inferior horn of the lateral ventricle, severe whole‐brain volume reduction, and longer disease duration. Although cortical atrophy was significantly more common in the progressive phase, approximately half of the MS patients with the severe cortical atrophy pattern had relapsing–remitting disease.

**Conclusion:**

Cortical thickness reduction patterns in MS are mostly characterized by the degree of temporal lobe cortical atrophy, which may start in the relapsing–remitting phase. Among the temporal lobe cortices, the neurodegenerative change may accelerate in the temporal pole in the progressive phase.

## Graphical abstract

MS patients can be classified into three cortical thickness reduction patterns (CPs) that reflect the degree of global cortical atrophy with some uneven distribution. The most significant difference among CPs is the degree of temporal cortical atrophy and only when temporal cortical atrophy becomes severe is temporal pole atrophy observed. Temporal pole atrophy is associated with demyelinating plaques in adjacent areas.

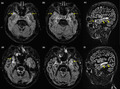

## 1 INTRODUCTION

Multiple sclerosis (MS) is a chronic inflammatory demyelinating and neurodegenerative disease of the central nervous system (Reich et al., 2018). Brain atrophy is often seen at disease presentation, and current data suggest that it proceeds inexorably—even in progressive disease when new inflammatory lesions are rare (Beck & Reich, 2018). The assessment of neurodegeneration in terms of brain atrophy, particularly gray matter atrophy, is of high clinical relevance because it has substantial predictive value with respect to long‐term physical disability, cognitive decline, and disease progression (Calabrese et al., 2010; Filippi et al., 2013; Rocca et al., 2017; Sastre‐Garriga et al., 2020; Vigeveno et al., 2012).

Although previous studies had indicated widespread cortical atrophy in MS (Kim et al., 2016; Liu et al., 2014), a recent cross‐sectional study suggested that cortical gray matter atrophy in MS occurs mainly in a nonrandom manner and identified six cortical atrophy patterns. The identified atrophy patterns showed more pronounced cortical atrophy in the posterior cingulate cortex and the temporal pole (Steenwijk et al., 2016). Additionally, the latter pattern exclusively showed significantly more atrophy in secondary‐progressive (SP) MS than in relapsing–remitting (RR) MS (Steenwijk et al., 2016). However, few studies have identified patterns of cortical gray matter atrophy in MS. In contrast, in other neurodegenerative diseases such as Alzheimer's disease and Parkinson's disease, cluster analyses using the brain MRI volume data of patients' cortical thickness have identified subtypes with distinct clinical features in each of these diseases (Noh et al., 2014; Uribe et al., 2016).

Thus, our study aimed to investigate nonrandom patterns of cortical thickness reduction, which might be not fully resolved, in Japanese MS patients. Since we had previously identified several brain volume loss patterns in MS patients by performing a hierarchical cluster analysis on regional brain volume data (Fujimori, et al., 2020), we tried to examine cortical thickness reduction patterns in this study by performing the same analysis on brain cortical thickness data in the same MS cohort as in our prior research.

## 2 METHODS

### 2.1 Patients

Seventy‐five consecutive MS patients (3 patients with CIS, 59 patients with RRMS, and 13 patients with progressive MS) and 21 consecutive healthy controls (HCs) were recruited cross‐sectionally at the Division of Neurology at Tohoku Medical and Pharmaceutical University in Sendai, Japan, between June 2017 and June 2018, as previously described (Fujimori et al., 2019). Progressive MS that consists of primary progressive MS and SPMS were identified by a history of progressive accrual of disability independent of relapse (Ontaneda, 2019). Among the 75 MS patients, one patient with RRMS was excluded from this study since one parameter of the cortical thickness data could not be obtained by FreeSurfer analysis. The inclusion criteria were as follows: (1) MS diagnosed by an experienced MS neurologist (IN) according to the 2017 revisions of the McDonald criteria (Thompson et al., 2018), (2) age between 20 and 70 years, and (3) no history of relapse in the last 3 months. The exclusion criteria were as follows: (1) neuromyelitis optica spectrum disorders (NMOSDs) or myelin oligodendrocyte glycoprotein antibody‐associated disorders, (2) a history of psychiatric illness other than stable depressive symptoms, (3) a history of developmental delay, and (4) major medical conditions. We used the Expanded Disability Status Scale (EDSS) (Kurtzke, 1983) to measure the disability of the patients. The local institutional ethics committee at Tohoku Medical and Pharmaceutical University approved the study protocol. Written informed consent was obtained from all participants.

### 2.2 MRI acquisition

All study subjects were scanned on the same whole‐body MRI system operating at 1.5 Tesla (MAGNETOM Aera, Siemens, Germany, HE1‐4 coil) using a standardized acquisition protocol, including a high‐resolution sagittal 3‐dimensional (3D) T1‐weighted magnetization‐prepared rapid gradient‐echo (MPRAGE) sequence (repetition time (TR): 2,730 ms, echo time (TE): 3.3 ms, inversion time (TI): 1,000 ms, 176 slices, field of view (FoV): 256 mm, measured isotropic voxel size: 1 × 1 × 1 mm). Additionally, the protocol included a sagittal 3D FLAIR sequence (TR: 5,000 ms, TE: 335 ms, TI: 1,800 ms, 176 slices, FoV: 256 mm, measured isotropic voxel size: 1 × 1 × 1 mm) and a sagittal 3D double inversion recovery (DIR) sequence (TR: 7,500 ms, TE: 309 ms, TI_1_: 3,000 ms, TI_2_: 450 ms, 120 slices, FoV: 256 mm, measured isotropic voxel size: 1.3 × 1.3 × 1.5 mm).

### 2.3 MRI postprocessing to measure global and regional brain volumes

The 3D‐MPRAGE dataset served as the input data for the postprocessing pipeline. The cortical thickness of a total of 68 regions of interest and regional and whole‐brain volumes were estimated using the automated FreeSurfer stream (version 5.3.0, http://surfer.nmr.harvard.edu) as previously described (Dale et al., 1999; Dale & Sereno, 1993; Fischl & Dale, 2000; Fischl et al., 2001, 2002; Fujimori et al., 2019, 2020; Segonne et al., 2007). The procedures performed by FreeSurfer included the removal of nonbrain data, intensity normalization (Fischl et al., 2001), tessellation of the gray matter/white matter boundary, automated topology correction (Dale et al., 1999; Segonne et al., 2007), and accurate surface deformation to identify tissue borders (Dale & Sereno, 1993; Fischl & Dale, 2000; Fischl et al., 2002). The Desikan–Killiany Atlas, consisting of 34 regions per hemisphere, was employed to determine average cortical thickness in each area (Desikan et al., 2006). After FreeSurfer preprocessing, the results for each subject were visually inspected by an experienced reader (JF) to ensure accuracy of registration, skull stripping, segmentation, and cortical surface reconstruction. Possible errors were fixed by manual intervention. We used raw cortical thickness data for further analysis. The brain volume data were directly extracted for a total of 56 segments obtained for each patient from FreeSurfer's automated segmentation results, as described in our prior research (Fujimori, et al., 2020), and normalized to head size using the total intracranial volume (also obtained from FreeSurfer) (Azevedo et al., 2015; Rocca et al., 2017). These unitless values were used for further analysis.

### 2.4 Cluster analysis using cortical thickness data

We performed a hierarchical cluster analysis using brain MRI cortical thickness data to divide the MS patients into several clusters. Each cluster included patients who shared a similar cortical thickness reduction pattern. Clustering is a multivariate technique that groups together observations that share similar values across multiple variables (https://www.jmp.com/support/help/13‐2/Hierarchical_Cluster_Overview.shtml). We used Ward's clustering linkage method to combine clusters (Ward, 1963). The cluster analysis was performed using the 68 regions of interest in the cortical gray matter obtained from each of the 74 subjects with MS.

### 2.5 Principal component analysis (PCA) of cortical thickness

PCA was used to validate the clustering results (Whitwell et al., 2009) as previously described (Fujimori, et al., 2020). PCA is a technique by which a high‐dimensional dataset is projected onto a lower‐dimensional (uncorrelated) space (Whitwell et al., 2009). This method is used to model the variation in a set of variables in terms of a smaller number of independent linear combinations (*principal components*) of those variables (https://www.jmp.com/support/help/13‐2/Overview_of_Principal_Component_Analysis.shtml). The first two principal components can be interpreted as the best two‐dimensional representation of the full dataset, capturing as much variability as possible (Whitwell et al., 2009). We performed PCA using the 68 regions of interest in the cortical thickness obtained for each of the 74 subjects with MS. The hierarchical cluster analysis and PCA were performed using the software JMP, version 13.0 (SAS Institute Inc., Cary, NC, USA).

### 2.6 Lesion volumetry by Icometrix

The 3D FLAIR and 3D T1 MPRAGE datasets obtained in each patient were analyzed using the program MSmetrix by uploading the DICOM data to the Icometrix website (http://icometrix.com) as previously described (Akaishi et al., 2017; Jain et al., 2015).

### 2.7 MRI postprocessing to measure the volumes of DIR high‐intensity lesions

DIR images were postprocessed by using an independent 3D volume‐analyzer workstation (SYNAPSE 3D; Japanese local name, SYNAPSE VINCENT; Fujifilm Medical Systems, Tokyo, Japan) (https://www.fujifilmusa.com/products/medical/medical‐informatics/radiology/3D/). Hyperintense lesions on DIR images in the cortical gray matter and adjacent juxtacortical white matter were identified based on the recommended definitions for cortical lesion scoring and classification (Geurts et al., 2011). Multiple slices, including axial and sagittal images, were viewed to determine lesion distribution. The volumes of high‐intensity lesions on DIR images were measured by manually enclosing each lesion as a region of interest.

### 2.8 Statistical analysis

Statistical analyses were performed using JMP version 13.0 software. Comparisons of clinical or MRI data between the MS patients and the HCs were evaluated via Pearson's chi‐square test or the Wilcoxon test. Multiple comparisons of cortical thickness, clinical data, brain volume, or lesion loads among the MS clusters and HCs were performed using the Steel–Dwass test, controlling for the overall experiment‐wise error rate, or Pearson's chi‐square test. Correlations between cortical thickness and brain volume or lesion loads were evaluated using a density ellipsoid at a probability level of 0.95. Statistical significance was defined using an *α*‐level of 0.05, which, after the Bonferroni correction with a factor of 70 for multiple comparisons, was equivalent to 0.0007 for this hypothetical exploratory study. Intrarater agreement analysis was performed for DIR high‐intensity lesion loads using the intraclass correlation coefficient (ICC). ICC values of <0.40 were considered poor, 0.40‐0.75 were fair to good, and >0.75 were excellent based on statistical conventions (Granberg et al., 2015).

## 3 RESULTS

### 3.1 Patient clinical profiles

Seventy‐four MS patients (females/males = 55/19) were included in this study. The study cohort consisted of patients presenting with clinically isolated syndrome (CIS) (4%), RRMS (79%), and progressive MS (17%). The mean age of the patients was 40.3 ± 9.9 years, and the mean duration of disease was 9.6 ± 7.3 years. The median EDSS score was 2 (1–3.5), and 64 of the MS patients (86.5%) were treated with disease‐modifying therapy (DMT). Ten patients were treated with interferon beta (13.5%); 38, fingolimod (51%); 11, dimethyl fumarate (15%); and 5, other drugs (7%) (2 patients received glatiramer acetate, 2 patients received natalizumab, and one patient was in a clinical trial (ofatumumab)). The mean age of the HCs (female/male = 11/10) was 36.2 ± 13.6 years. Conventional brain MRI scans revealed no abnormal findings in the HCs. Sex distribution and age did not significantly differ between the MS and HC groups. As the distribution of age did not significantly differ between the two groups, it was not taken into account when comparing volumes. The mean lesion load in MS patients was 7.35 ± 7.74 ml. Mean volumes of the whole brain, cortical white matter, and total gray matter were all significantly reduced in MS patients compared with HCs (Table 1).

**Table 1:** Clinical profiles

Clinical profiles	MS (*n* = 74)	HCs (*n* = 21)	Comparisons between MS patients and HCs (*p*‐value)
Sex (F:M)	55:19	11:10	.054^a^
CIS	*n* = 3 (4%)		
RRMS	*n* = 58 (79%)		
PMS	*n* = 13 (17%)		
Age, mean (*SD*)	40.3 (9.9)	36.2 (13.6)	.114^b^
Duration (years), mean (*SD*)	9.6 (7.3)		
EDSS score, median (IQR)	2 (1–3.5)		
Interferon beta	*n* = 10 (14%)		
Fingolimod	*n* = 38 (51%)		
Dimethyl fumarate	*n* = 11 (15%)		
Glatiramer acetate	*n* = 2 (3%)		
Natalizumab	*n* = 2 (3%)		
Ofatumumab	*n* = 1 (1%)		
Lesion volume, mean (*SD*) (ml)	7.35 (7.74)		
Brain segmentation vol, mean (*SD*) (ml)	1,038,836 (111,310)	1,127,191 (121,547)	.005^b^
Cortical white matter vol, mean (*SD*) (ml)	420,143 (59,029)	466,172 (64,649)	.004^b^
Total gray matter vol, mean (*SD*) (ml)	563,525 (60,249)	613,065 (61,480)	.003^b^

Note

Brain segmentation volume represents whole‐brain volume.

Abbreviations: CIS, clinically isolated syndrome; EDSS, Expanded Disability Status Scale; HC, healthy control; PMS, progressive MS; RRMS, relapsing–remitting MS; Vol, volume.

^a^ *p*‐value determined via Pearson's chi‐square test.

^b^ *p*‐value determined through the Wilcoxon test.

### 3.2 Cluster analysis identified three cortical thickness reduction patterns based on cortical thickness data

**FIGURE 1:**
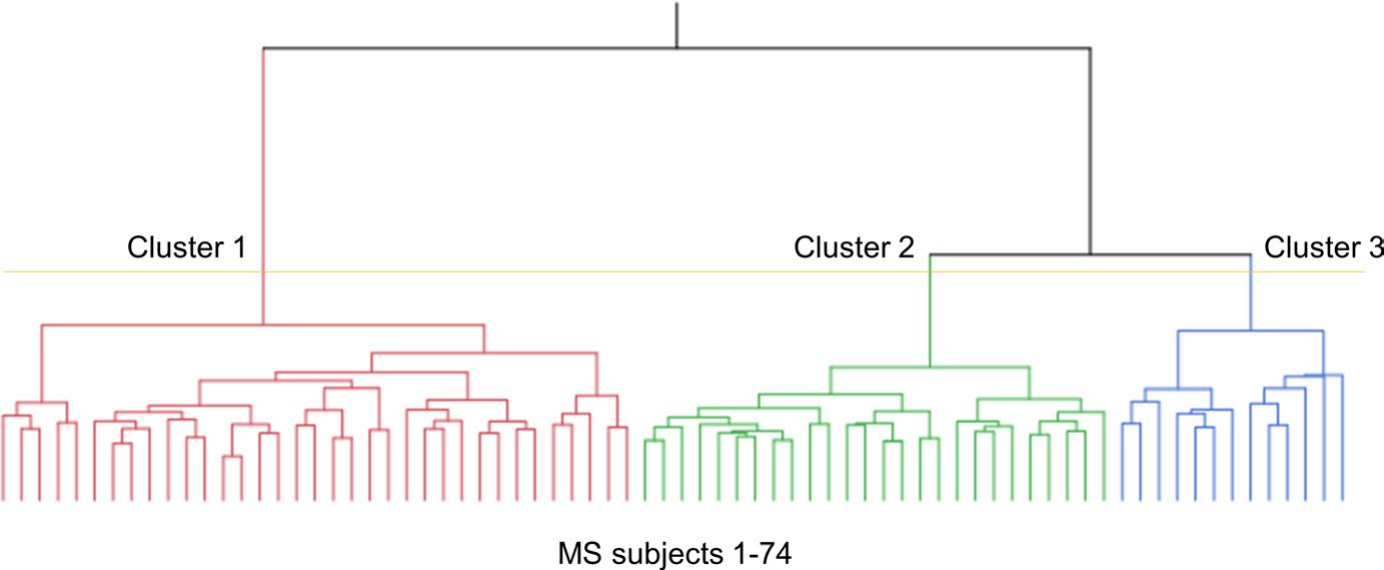
Dendrogram of multiple sclerosis (MS) patients clustered according to cortical thickness data. The distance along the y‐axis represents the similarity among the clusters: A shorter distance indicates a greater degree of similarity. The horizontal axis represents the 74 MS patients included in the cluster analysis. At the 3‐cluster level (yellow line), MS patients were clustered into three subgroups: Cluster 1 (C1), Cluster 2 (C2), and Cluster 3 (C3)

**FIGURE 2:**
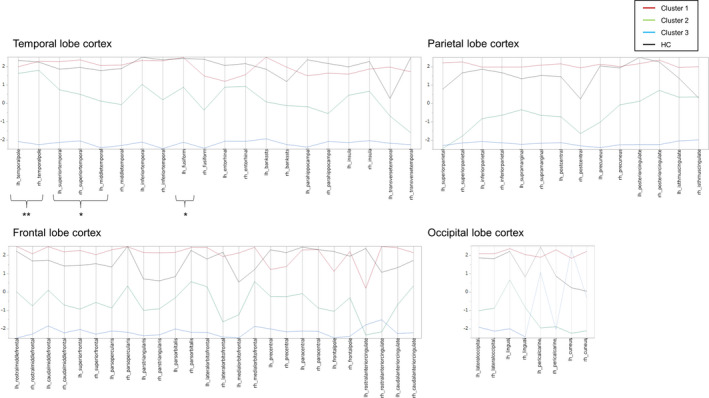
Profile of cortical thickness at the 3‐cluster level. A parallel plot shows the mean cortical thicknesses of the three clusters. The thickness of 68 samples of cortical gray matter is shown under four classifications (the temporal, frontal, parietal, and occipital cortexes) (https://surfer.nmr.mgh.harvard.edu/fswiki/CorticalParcellation). The mean cortical thickness was standardized by subtracting the mean and dividing by the standard deviation of the cortical thicknesses of all the patients, and the standardized mean cortical thickness of each cluster was plotted. The *y*‐axis for each variable extends two standard deviations above and below the mean. *Most of the global cortical thicknesses were equivalent between the HCs and Cluster 1, whereas they were significantly different among the three clusters; the included regions encompassed the bilateral superior and left middle temporal cortexes, as well as the left fusiform cortex. **In contrast, the cortical thickness of the temporal pole was significantly different between Cluster 3 and Cluster 1, Cluster 2 and the HC group. Temporal pole: temporal pole; superior temporal: superior temporal gyrus; middle temporal: middle temporal gyrus; inferior temporal: inferior temporal gyrus; fusiform: fusiform gyrus; entorhinal: entorhinal cortex; BANKSSTS: banks of the superior temporal sulcus; parahippocampal: parahippocampal gyrus; transverse temporal: transverse temporal cortex; rostral middle frontal: rostral middle frontal gyrus; caudal middle frontal: caudal middle frontal gyrus; superior frontal: superior frontal gyrus; parsopercularis: pars opercularis; parstriangularis: pars triangularis; parsorbitalis: pars orbitalis; lateral orbitofrontal: lateral orbital frontal cortex; medial orbitofrontal: medial orbital frontal cortex; precentral: precentral gyrus; paracentral: paracentral lobule; frontal pole: frontal pole; rostral anterior cingulate: rostral anterior cingulate; caudal anterior cingulate: caudal anterior cingulate; superior parietal: superior parietal cortex; inferior parietal: inferior parietal cortex; supramarginal: supramarginal gyrus; postcentral: postcentral gyrus; precuneus: precuneus cortex; posterior cingulate: posterior cingulate; isthmus cingulate: isthmus cingulate; lateraloccipital: lateral occipital cortex; lingual: lingual gyrus; pericalcarine: pericalcarine cortex; cuneus: cuneus cortex

The cluster analysis results are shown as a dendrogram (Figure 1). At the 3‐cluster level, the 74 MS patients were divided into three cortical thickness reduction patterns (Clusters 1, 2, and 3). At the 4‐cluster level, one cluster consisted of a small number of patients. Hence, we selected the 3‐cluster level to classify patients into subgroups to establish clinical significance in our subsequent analyses. A parallel plot of the cluster means showed that there were clear differences among all of the cortical thickness reduction patterns (Figure 2). As outlined in Table S1 and Figure 2, most of the regional cortical thicknesses were equivalent between the HCs and Cluster 1, but there were significant differences among the three clusters in certain regions, including the bilateral superior and left middle temporal cortexes, as well as the left fusiform cortex. In contrast, a significant decrease in the cortical thickness of the bilateral temporal pole was observed only in Cluster 3, whereas there were no significant differences among Cluster 1, Cluster 2, and the HC group. In addition, a significant difference between the HCs and patients in Cluster 3 was observed bilaterally in the temporal pole; the superior, middle, and inferior temporal cortexes; the fusiform cortex; and the rostral middle frontal and precentral cortex (Table S1). In contrast, the thicknesses of the pericalcarine and rostral anterior cingulate cortexes signalled the least significant difference among Cluster 1, Cluster 2, Cluster 3, and the HC group (Table S1).

### 3.3 PCA

**FIGURE 3:**
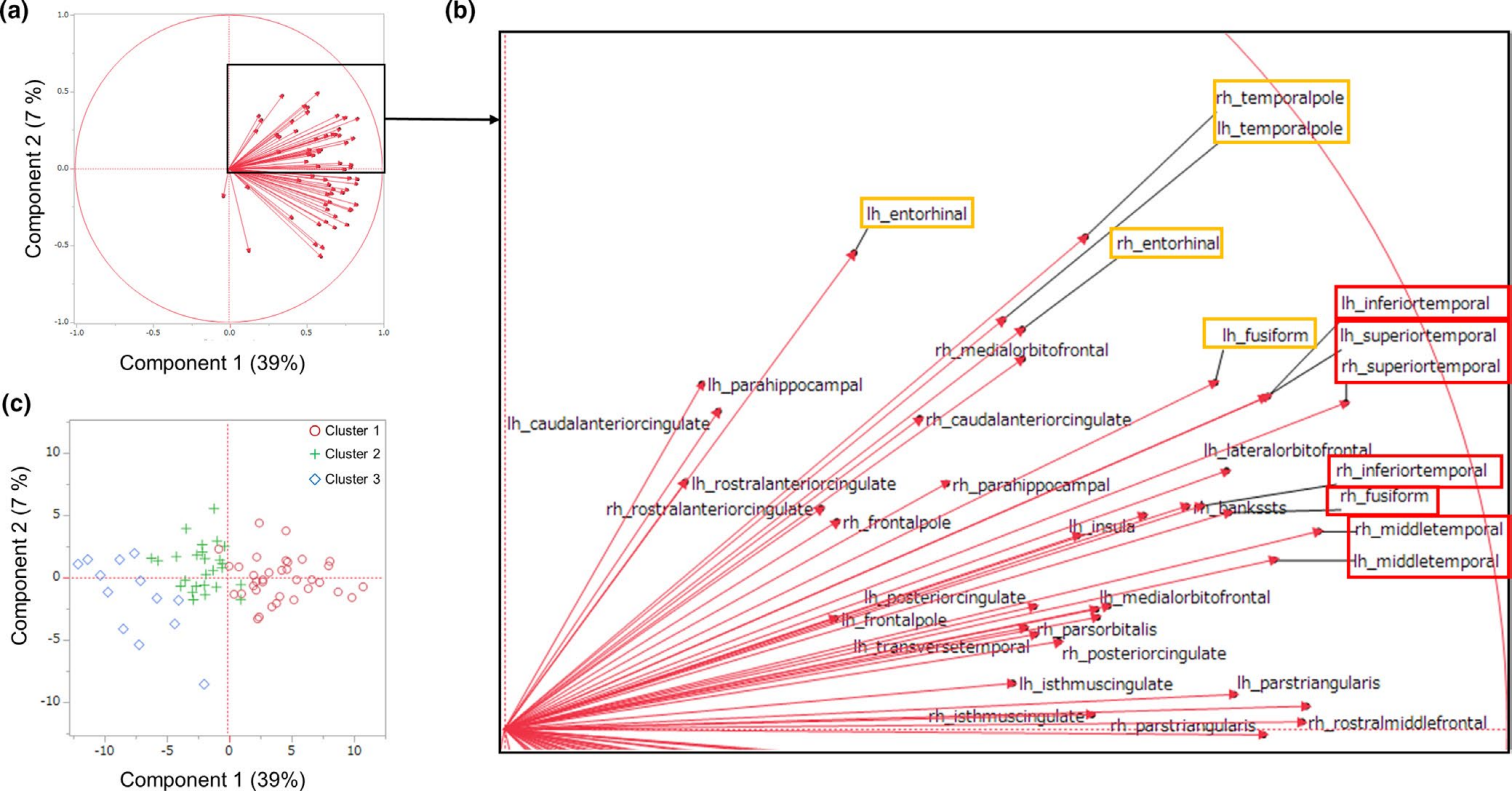
The separation of the three MS subtypes based on the first and second principal components. PCA was used to obtain the first and second principal components (components 1 and 2), which can be interpreted as the best two‐dimensional representation of the full, high‐dimensional dataset of cortical thickness. The contributions of components 1 and 2 to the full dataset were 39% and 7%, respectively. (a) Loading plots showing the loadings of variables (68 samples of brain cortical thickness) for each component (the first and second principal components). As the value approaches 1, the effect of the component on that variable increases. The first principal component (component 1, horizontal axis) positively represents the cortical thickness of most regions, including the bilateral superior, middle, and inferior temporal cortex. (b) Enlargement of the box in Figure (a). The second principal component positively represented the bilateral temporal pole, the supra temporal cortex, the entorhinal cortex, the left inferior temporal cortex, the left fusiform cortex, the right medial orbitofrontal cortex, and the right caudal anterior cingulate. (c) Score plot graphs showing each component's calculated values in relation to the other, with each value adjusted for the mean and standard deviation. Patients who belonged to Clusters 1 (red circles), 2 (green plus signs), and 3 (blue diamonds) due to the cluster analysis were separated from one another by combining the first and second principal components

Using PCA, we obtained the first and second principal components (components 1 and 2). The contributions of components 1 and 2 to the variance of the full dataset were 39% and 7%, respectively. The first principal component positively represented the cortical thickness of various regions including the bilateral superior, middle, and inferior temporal cortex (Figure 3a,b). The second principal component positively represented the bilateral temporal pole, the superior temporal cortex, the entorhinal cortex, the left inferior temporal cortex, the left fusiform cortex, the right medial orbitofrontal cortex, and the right caudal anterior cingulate (Figure 3b). A score plot of the PCA was used to plot the calculated values from each component (components 1 and 2) in relation to each other. Patients who belonged to Clusters 1, 2, or 3 due to the cluster analysis were separated from each other by combining the first and second principal components (Figure 3c).

### 3.4 Comparisons of clinical profiles among the patients with the three cortical thickness reduction patterns and the HCs

Cluster 1 (*n* = 35) consisted of patients with CIS (*n* = 3), RRMS (*n* = 31), and progressive MS (*n* = 1). Cluster 2 (*n* = 26) was made up of patients with RRMS (*n* = 21) and progressive MS (*n* = 5). Cluster 3 (*n* = 13) was comprised of patients with RRMS (*n* = 6) and progressive MS (*n* = 7). The chi‐square test revealed a significant difference in the proportions of these subtypes among the three clusters (*p* = .0015). Patient sex did not significantly differ among the 3 clusters and the HC group. Patient age was significantly older in Cluster 2 (*p* = .0033) and Cluster 3 (*p* = .0028) than in Cluster 1. Disease duration was significantly longer in Cluster 3 than in Cluster 1 (*p* = .0033) and Cluster 2 (*p* = .0357). The EDSS scores were significantly lower in Cluster 1 than in Cluster 2 (*p* = .0042) and Cluster 3 (*p* = .0005). The numbers of patients treated with DMT in Cluster 1, Cluster 2, and Cluster 3 were 30 (86%), 22 (85%), and 12 (92%), respectively, and there was no significant difference among the 3 clusters. The drugs used for DMT also did not significantly differ among the 3 clusters.

We classified the MS patients into three major subgroups (mild, moderate, and severe brain volume loss (BVL) groups) according to their brain volume patterns using a hierarchical cluster analysis with multivariate brain volume data as described in our previous study (Fujimori et al., 2019). We compared the findings of the present study with those of the previous one, since the two were conducted with the same subjects. Among the 35 patients in Cluster 1, 28 (80%) belonged to the mild BVL subgroup, and 7 (20%) belonged to the moderate BVL subgroup. Among the 26 patients in Cluster 2, 9 (35%) belonged to the mild BVL subgroup, 16 (62%) belonged to the moderate BVL subgroup, and one (3%) belonged to the severe BVL subgroup. Among the 13 patients in Cluster 3, 2 (15%) belonged to the moderate BVL subgroup, and 11 (85%) belonged to the severe BVL subgroup.

### 3.5 Comparison of the lateral ventricle volume and lesion loads among the three clusters and the HCs

**FIGURE 4:**
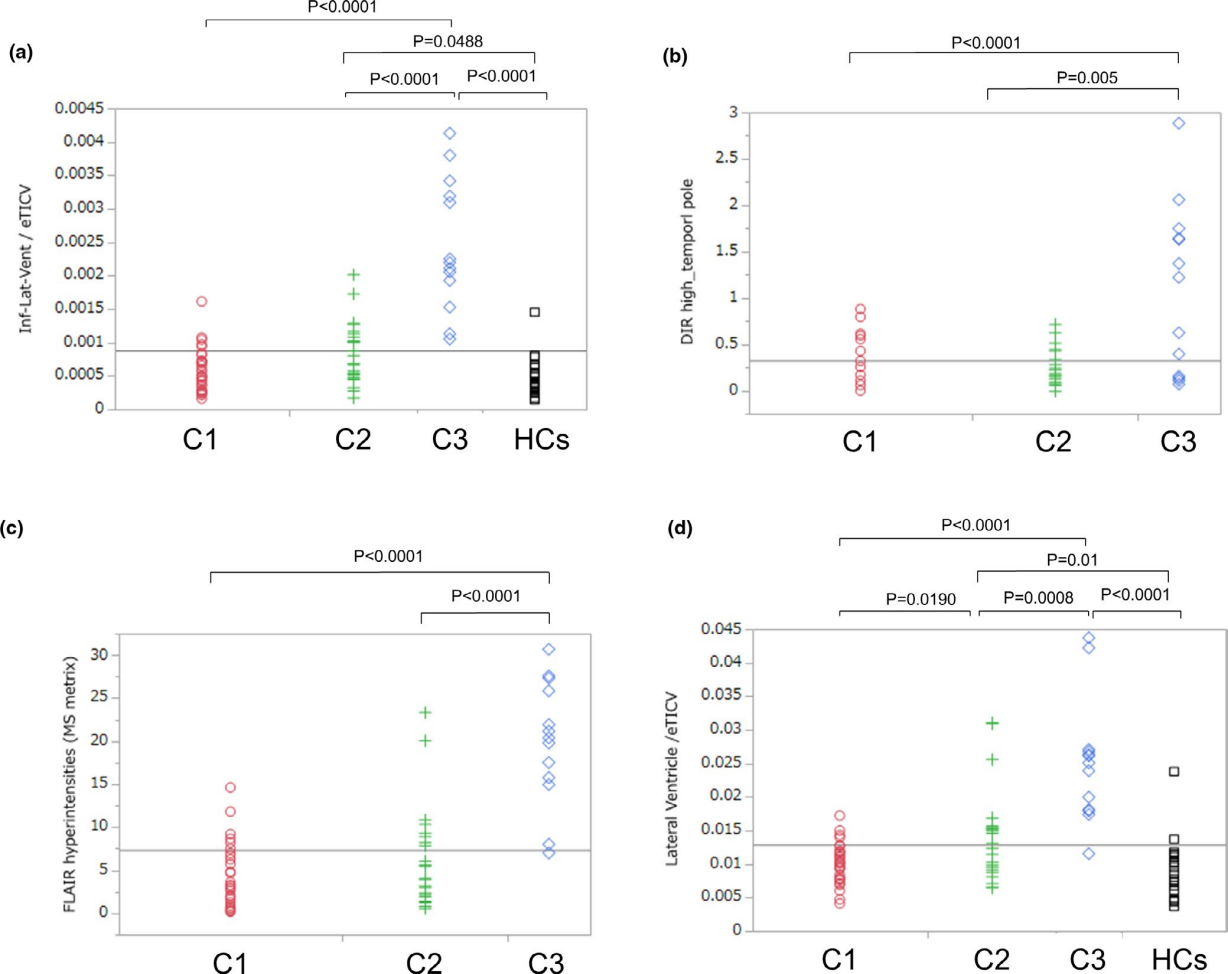
Comparisons of regional volumes and lesion loads among the three clusters and the HC group. (a) The sum of the normalized volume of the left and right inferior horn of the lateral ventricle was significantly increased in Cluster 3 relative to that of Cluster 1 (*p* < .0001) and Cluster 2 (*p* < .0001), whereas there were no significant differences between Clusters 1 and 2. (b, c) The DIR high‐intensity lesion loads in the temporal pole and the adjacent juxtacortical white matter, as well as the total FLAIR high‐intensity lesion loads, were also significantly increased in Cluster 3 relative to Clusters 1 (*p* < .0001) and 2 (*p* = .005 and *p* < .0001, respectively), whereas there were no significant differences between Clusters 1 and 2. (d) In contrast, the sum of the normalized volumes of the left and right lateral ventricles were significantly increased in Cluster 2 relative to Cluster 1 (*p* = .0190), although they were also significantly increased in Cluster 3 relative to Cluster 1 (*p* < .0001) and Cluster 2 (*p* = .0008). C1: Cluster 1, C2: Cluster 2, C3: Cluster 3, HCs: healthy controls

**FIGURE 5:**
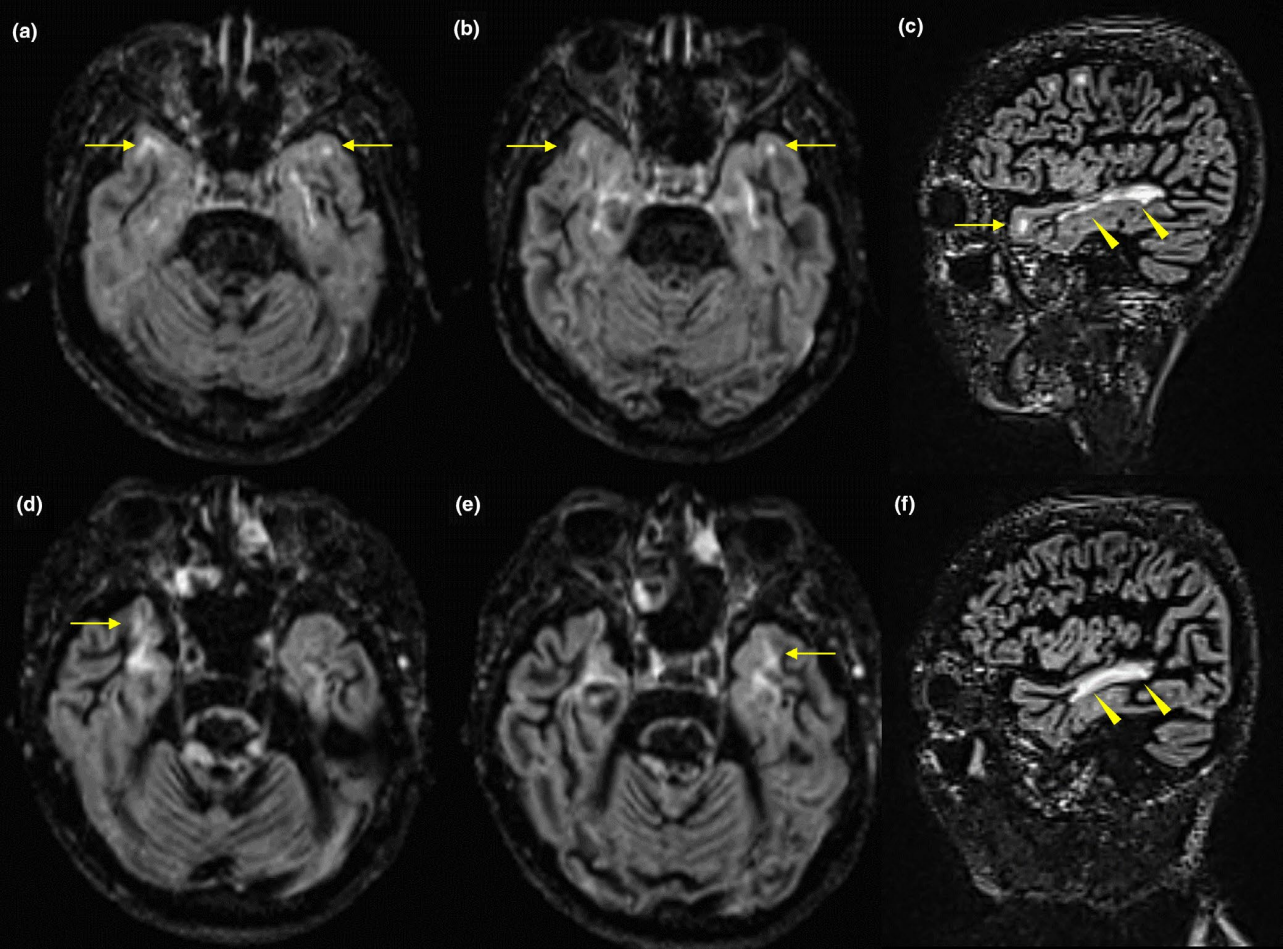
Representative brain MRI scans in MS patients with Cluster 3. Axial and sagittal 3D double inversion recovery (DIR) sequences (TR: 7,500 ms, TE: 309 ms, TI 1:3,000 ms, TI 2:450 ms, 120 slices, FoV: 256 mm, measured isotropic voxel size: 1.3 × 1.3 × 1.5 mm) are shown. (a‐c) A 38‐year‐old female RRMS patient with a disease duration of 24 years and an EDSS score of 2 was classified under the Cluster 3. (d‐f) A 41‐year‐old male SPMS patient with a disease duration of 20 years and an EDSS score of 8 was categorized into the Cluster 3. Both patients presented with cognitive impairments. Axial and sagittal DIR images showed high‐intensity lesions in the temporal pole and in the adjacent juxtacortical white matter (arrows) (a, b, c, d, e). Sagittal DIR images showed white matter lesions in the temporal lobe (arrow heads) (c, f)

Since we identified the temporal pole as a characteristic cortical gray matter region specifically atrophied in Cluster 3, we further examined whether temporal pole thickness reductions reflected degenerative change, which we evaluated by the degree of dilation in the inferior horn of the lateral ventricle, which is located close to the temporal pole (Kiernan, 2012). In this post hoc analysis, we compared the volumes of the lateral ventricle, the inferior horns of the lateral ventricle, the DIR high‐intensity lesion loads, and the total FLAIR high lesion loads among Clusters 1, 2, 3, and the HCs. Compared with those in Clusters 1 and 2, the sum of the normalized volumes of the left and right inferior horns of the lateral ventricle were significantly increased in Cluster 3, whereas there were no significant differences between Clusters 1 and 2 (Figure 4a). The intrarater ICC was 0.87 for the DIR high‐intensity lesion loads. The DIR high‐intensity lesion loads in the temporal pole and the adjacent juxtacortical white matter (Figure 5), as well as the total FLAIR high lesion loads, were also significantly increased in Cluster 3 with respect to Clusters 1 and 2, whereas there were no significant differences between Clusters 1 and 2 (Figure 4b,c). In contrast, the sum of the normalized volumes of the left and right lateral ventricles were significantly increased in Cluster 2 with respect to Cluster 1, although they were also significantly increased in Cluster 3 with respect to Clusters 1 and 2 (Figure 4d). Therefore, an increase in the normalized volume of the inferior horn of the lateral ventricle (not the lateral ventricle), the DIR high‐intensity lesion loads in the temporal pole and the adjacent juxtacortical white matter, and the total FLAIR high lesion loads, as well as a decrease in the thickness of the temporal pole, were all specifically observed in Cluster 3.

### 3.6 The relationship between temporal pole thickness and lateral ventricle volume

In this post hoc analysis, we scrutinized the relationship between temporal pole thickness and lateral ventricle volume. The mean thicknesses of the left and right temporal poles among the 74 MS patients were 3.363 ± 0.489 and 3.497 ± 0.487, respectively (Table 2). The thickness of the left and right temporal poles was more significantly correlated with the normalized volume of the ipsilateral inferior horn of the lateral ventricle than with the normalized volume of the ipsilateral lateral ventricle. The results were essentially the same when we analyzed the non‐normalized volume of the inferior horn of the lateral ventricle and the lateral ventricle.

**Table 2:** The relationship between temporal pole thickness and the lateral ventricle volumes in MS patients

	lt temporal pole thickness	rt temporal pole thickness
Ipsilateral lateral ventricle*/TICV	*p *< .0001 (*r* = −.452)	*p *< .0001 (*r* = −.549)
Ipsilateral inferior lateral ventricle*/TICV	*p *< .0001 (*r* = −.504)	*p *< .0001 (*r* = −.673)

Abbreviation: TICV, total intracranial volume.

* Volumes of the lateral ventricle and the inferior horn of the lateral ventricle on the same side as the temporal pole.

### 3.7 The relationship between temporal pole thickness and lesion loads

In this post hoc analysis, we also looked at whether temporal pole thickness reduction reflected demyelinating lesion loads in the temporal pole and the adjacent juxtacortical white matter. The sum of the mean thickness of the left and right temporal poles among the MS patients was 6.860 ± 0.900 (Table 3). The sum of the thickness of the left and right temporal poles was more significantly correlated with the volume of the DIR high‐intensity lesion loads of the bilateral temporal poles and the adjacent juxtacortical white matter than with the volume of the total lesion loads seen in FLAIR images.

**Table 3:** The relationship between temporal pole thickness and lesion loads in MS patients

	Temporal pole thickness
Total FLAIR hyperintensities (7.354 ± 7.742)	*p *< .0001 (*r* = −.633)
DIR hyperintensities in temporal pole (0.328 ± 0.549)	*p *< .0001 (*r* = −.735)

Note

Total FLAIR hyperintensities: Total FLAIR high‐intensity lesion loads in the entire brain.

DIR hyperintensities in the temporal pole: DIR high‐intensity lesion loads in the bilateral temporal poles and the adjacent juxtacortical white matter.

## 4 DISCUSSION

In this study, we showed that MS patients present several cortical thickness reduction patterns, mostly reflecting global cortical gray matter thickness reduction. Among these cortical gray matter regions, only temporal lobe gray matter (the bilateral superior and left middle temporal cortex, as well as the left fusiform cortex) exhibited significant differences in thickness between the MS patients with three cortical thickness reduction patterns and the HCs. We also found that the temporal pole is a rather characteristic anatomical area, with pronounced cortical gray matter atrophy, particularly in those patients with a severe global reduction in cortical thickness. PCA also confirmed that the thickness of these temporal lobe cortexes significantly contributes to dividing MS patients based on the three cortical thickness reduction patterns. Further, in the post hoc analysis, dilations of the inferior horn of the lateral ventricle and lesion loads in the cortical gray matter of the temporal pole and the adjacent juxtacortical white matter were also more frequently observed in patients with severe global cortical thickness reduction. Most MS patients with severe global cortical thickness reduction presented severe global brain volume loss and longer disease duration and tended to have progressive MS more often than patients with less severe reductions. These results indicated that cortical gray matter atrophy in MS is well categorized by the progression of temporal lobe cortical atrophy. Moreover, temporal pole thickness reduction is one of the characteristic late neurodegenerative changes reflecting focal demyelination and neurodegeneration, which is observed specifically in MS patients who have developed severe volume loss of the whole‐brain and cortical gray matter. Severe cortical gray matter thickness reductions were more frequently identified in progressive MS than in other disease stages, as reported in previous studies (Eshaghi et al., 2018; Steenwijk et al., 2016).

It remains unclear whether cortical gray matter atrophy in MS is a result of diffuse neurodegeneration or develops according to distinct anatomical patterns. Steenwijk and colleagues cross‐sectionally searched for anatomical patterns of covarying cortical thicknesses using source‐based morphometry in MS and identified six cortical atrophy patterns, of which those with the most pronounced cortical atrophy were located in the bilateral posterior cingulate cortex and the bilateral temporal pole. They concluded that cortical atrophy in MS occurs largely in a nonrandom manner and develops (at least partly) according to distinct anatomical patterns (Steenwijk et al., 2016). Our study showed that the pattern of cortical thickness in MS patients could be categorized into three main clusters based on cluster analysis. Although the categorization reflected the degree of global cortical thickness, the degree of cortical thickness reduction was different depending on the anatomical site. The cortical gray matter areas that had the most significant differences between those with the severe global cortical thickness reductions and HCs also included the temporal pole.

Recently, Eshaghi and colleagues longitudinally investigated whether there is a spatiotemporal pattern of gray matter atrophy in MS and reported that volume loss of the whole cortical gray matter was faster in patients with SPMS (−1.11% per year), PPMS (−0.79%), and RRMS (−0.67%) than in HCs (−0.34%). Among the cortical regions, the temporal lobe showed faster gray matter volume loss in SPMS (−1.21%) than in RRMS (−0.77%) and CIS (−0.75%), while among the temporal lobe regions showing gray matter loss, the fastest volume loss was observed in the temporal pole (−1.47%) in SPMS (Eshaghi et al., 2018). Steenwijk and colleagues also reported that the bilateral temporal pole was the only cortical gray matter area among the 6 identified atrophied cortical gray matter areas that showed significantly more atrophy in secondary‐progressive than in relapsing–remitting patients (Steenwijk et al., 2016). Pathological studies have also demonstrated an increase in the rate of neurodegeneration, especially in the temporal regions, during progressive stages of MS in comparison with RRMS and CIS (Eshaghi et al., 2018; Haider et al., 2016; Howell et al., 2011; Kutzelnigg et al., 2005). These observations are supported by our study data, showing that the temporal pole was the only cortical gray matter area that presented with thickness reduction in the severe global cortical thickness reduction group but not in the mild or moderate cortical thickness reduction group. Since most of the MS patients classified as having severe global cortical thickness reduction presented with severe global brain volume loss and longer disease duration and more often had a progressive disease course than the patients in the other groups, our results were basically in accordance with those of previous studies.

The thickness of the pericalcarine and rostral anterior cingulate cortex were not significantly different among the MS patients within the three clusters and the HCs. These findings were basically in accordance with a previous study that reported that the occipital cortex showed a less significant difference in the rate of atrophy than the other cortexes among those with CIS, RRMS, SPMS, and PPMS (Eshaghi et al., 2018) and with another previous study that reported that the thickness of the anterior cingulate cortex was relatively preserved in MS relative to HCs (Steenwijk et al., 2016).

The temporal pole is part of the association cortex and is involved in multimodal sensory integration. It has been implicated in various higher order functions of socioemotional cognition, including language processing, face processing, emotion, and empathic behavior (Olson et al., 2007; Pehrs et al., 2017), although the exact function of the temporal pole remains unclear. However, these symptoms are rarely described as noticeable symptoms in MS patients, probably because atrophy of the temporal pole would be observed along with diffuse cortical atrophy in the progressive phase of the disease. In addition, atrophy of the temporal pole seemed to be much milder than that observed in neurodegenerative diseases. For example, the thickness of the temporal pole in patients with semantic variants of primary progressive aphasia was reported to be less than half that of the controls, whereas our data showed that the thickness of the temporal pole in Clusters 2 and 3 was approximately 96% and 70% that of the HCs, respectively (Collins et al., 2017). Further analysis is needed to clarify whether socioemotional cognition is affected in MS patients.

Interestingly, MS patients in Cluster 3 (*n* = 13) consisted of those with RRMS (*n* = 6) and progressive MS (*n* = 7). This outcome implied that the degree of cortical gray matter thickness reduction was not always consistent with clinical categorization. Although cortical gray matter thickness reductions in MS patients tended to become severe in progressive MS, other factors, including age, duration, and clinical phenotype, might contribute to this discordance. For example, among the cases diagnosed with SPMS, there may be cases in which EDSS has severely declined due to multiple spinal cord or brainstem lesions. Moreover, among the cases diagnosed with RRMS, there may be cases in which severe cortical atrophy has already occurred over time. These hypotheses might be supported by a recent concept, “silent progression,” which describes the insidious disability that accrues in many patients who satisfy traditional criteria for relapsing–remitting MS (University of California et al., 2019). Long‐term exacerbation is reported to be common in relapsing MS patients, is largely independent of relapse activity, and is associated with accelerated brain atrophy (University of California et al., 2019).

Our study has several limitations. First, this investigation is a single‐center study performed in a limited sample of Japanese MS patients; therefore, the results might have been influenced by selection bias. Furthermore, this categorization scheme may not be appropriate for other cohorts, since Japanese MS patients may present a slightly milder clinical course (Piccolo et al., 2015) and a slower rate of atrophy than those observed in Caucasian patients (Akaishi et al., 2017). However, there is general agreement that the Western‐type MS observed in Asia is not fundamentally different from that observed in typical MS in the Caucasian population once NMO and NMOSDs have been excluded (Polman et al., 2011). Moreover, although our MS patients consisted mostly of RRMS patients, the proportion of MS patients reflected that of the Japanese MS cohort as found in previous research (Houzen et al., 2018; Piccolo et al., 2015). Second, this investigation has a relatively small sample size for machine learning approaches, and the clusters may have been affected by a lack of patients representing early or late disease stages. Our results should be confirmed in other ethnic groups and in larger‐scale studies. In addition, latent profile analysis should be performed before hierarchical clustering to partially remove the resulting statistical bias. Third, DIR and FLAIR sequences have limited accuracy for the detection of MS lesions in the brain posterior fossa, especially at 1.5 T. In addition, the newest version of FreeSurfer is preferable for more accurate segmentation of cortical gray matter. Last, cognitive tests should be included in future research to assess the relationship between the identified clusters and cognitive functions.

In conclusion, we identified several cortical gray matter thickness patterns most significantly reflecting temporal lobe cortical thickness. In addition, temporal pole atrophy is one of the characteristic late neurodegenerative changes reflecting adjacent focal degeneration and demyelination that are specifically observed in MS with severe global brain and cortical atrophy. Furthermore, cortical gray matter thickness reduction in MS tended to become severe in progressive MS, although the degree of severity might not always be consistent with the clinical categorization, probably reflecting other factors, including patient age, disease duration, and clinical phenotype. The standardization and subsequent implementation of volumetric measurements for monitoring individual disease progression is one of the most relevant challenges in the near future, facilitating more accurate and individualized patient care.

## CONFLICT OF INTEREST

KF serves on scientific advisory boards for Bayer Schering Pharma, Biogen Idec, Mitsubishi Tanabe Pharma Corporation, Novartis Pharma, Chugai Pharmaceutical, Ono Pharmaceutical, Nihon Pharmaceutical, Merck Serono, Alexion Pharmaceuticals, MedImmune, and Medical Review; serves as an editorial board member for *Clinical and Experimental Neuroimmunology* (2009 to present) and an advisory board member for the *Sri Lanka Journal of Neurology*; and has received research support from Bayer Schering Pharma, Biogen Idec Japan, Asahi Kasei Medical, The Chemo‐Sero‐Therapeutic Research Institute, Teva Pharmaceutical, Mitsubishi Tanabe Pharma, Teijin Pharma, Chugai Pharmaceutical, Ono Pharmaceutical, Nihon Pharmaceutical, and Genzyme Japan. MW reports speaker or consultancy fees from Bayer Healthcare, Biogen, Biologix, Celgene, Eisai, Genilac, Imcyse, Merck Serono, Novartis, Roche, and Sanofi Genzyme. IN is serving on scientific advisory boards for Biogen Japan and Novartis Pharma and is receiving honoraria for speaking engagements with Biogen Japan, Mitsubishi Tanabe Pharma, Novartis Pharma, Takeda Pharmaceutical, and Eisai. JF reports no disclosure.

## AUTHOR CONTRIBUTIONS

All authors contributed to the study conception and design. Material preparation, data collection and analysis were performed by JF and IN. The first draft of the manuscript was written by JF, and all authors commented on previous versions of the manuscript. All authors read and approved the final manuscript.

## ETHICAL APPROVAL

This study was approved by the institutional ethics committee and has been performed in accordance with the ethical standards laid down in the 1964 Declaration of Helsinki and its later amendments.

## CONSENT TO PARTICIPATE

All patients provided written informed consent.

### PEER REVIEW

The peer review history for this article is available at https://publons.com/publon/10.1002/brb3.2050.

## Supporting information

Table S1

## Data Availability

Data are available upon reasonable request. Individual patient data will not be shared to conform with the privacy statement signed by the participants. Pseudonymized data may be shared upon request with the corresponding author.
