# Supplementary material for: Patterns of cortical grey matter thickness reduction in multiple sclerosis
Source: Brain Behav. 2021 Jan 27;11(4):e02050. doi: 10.1002/brb3.2050 (PMC8035454; doi:10.1002/brb3.2050)
Supplement: Supplementary file 1 — Table S1 [file BRB3-11-e02050-s001.docx]

**Table e-1. Comparison of the cortical thickness between three clusters and control.**

|  | **Cortical thickness ^a^, mean (SD)** | | | | **p value ^b^** | | | | | |
| --- | --- | --- | --- | --- | --- | --- | --- | --- | --- | --- |
|  | Cluster 1 (n=35) | Cluster 2 (n=26) | Cluster 3 (n=13) | Control (n=21) | C3 vs C2 | C3 vs C1 | C2 vs C1 | HC vs C3 | HC vs C2 | HC vs C1 |
| lh_MeanThickness | 2.39716 (0.055792) | 2.27810 (0.038571) | 2.17580 (0.062357) | 2.38353 (0.094469) | 0.0003 | <.0001 | <.0001 | <.0001 | 0.0003 | 0.9532 |
| rh_MeanThickness | 2.40154 (0.055442) | 2.28199 (0.038286) | 2.18567 (0.058690) | 2.38613 (0.083964) | 0.0003 | <.0001 | <.0001 | <.0001 | 0.0002 | 0.886 |
| lh_temporalpole | 3.53906 (0.387846) | 3.46385 (0.287576) | 2.69038 (0.505677) | 3.61167 (0.298159) | 0.0002 | 0.0002 | 0.5922 | <.0001 | 0.539 | 0.98 |
| rh_temporalpole | 3.71637 (0.221200) | 3.60623 (0.268144) | 2.68885 (0.529765) | 3.70752 (0.260937) | <.0001 | <.0001 | 0.5269 | <.0001 | 0.6948 | 0.9998 |
| lh_superiortemporal | 2.77411 (0.102778) | 2.61146 (0.127647) | 2.30646 (0.154428) | 2.73105 (0.110364) | <.0001 | <.0001 | <.0001 | <.0001 | 0.0056 | 0.5825 |
| rh_superiortemporal | 2.78403 (0.090852) | 2.60515 (0.101592) | 2.36346 (0.117681) | 2.74443 (0.106102) | 0.0002 | <.0001 | <.0001 | <.0001 | 0.0003 | 0.6365 |
| lh_middletemporal | 2.81997 (0.153100) | 2.66408 (0.106147) | 2.45992 (0.163987) | 2.79805 (0.094624) | 0.0005 | <.0001 | 0.0006 | <.0001 | 0.001 | 0.9731 |
| rh_middletemporal | 2.81349 (0.119630) | 2.66323 (0.109485) | 2.50769 (0.106745) | 2.80000 (0.110019) | 0.004 | <.0001 | 0.0001 | <.0001 | 0.0024 | 0.9835 |
| lh_inferiortemporal | 2.75251 (0.133157) | 2.64981 (0.106937) | 2.40392 (0.098153) | 2.76576 (0.158862) | <.0001 | <.0001 | 0.0198 | <.0001 | 0.0422 | 0.9937 |
| rh_inferiortemporal | 2.79100 (0.127938) | 2.68035 (0.095866) | 2.54215 (0.145922) | 2.79348 (0.123693) | 0.0139 | <.0001 | 0.0105 | 0.0004 | 0.0156 | 0.9977 |
| lh_fusiform | 2.61780 (0.103323) | 2.48885 (0.103193) | 2.25085 (0.188111) | 2.61395 (0.144824) | 0.0001 | <.0001 | 0.0001 | <.0001 | 0.0155 | 0.9745 |
| rh_fusiform | 2.62866 (0.116719) | 2.52158 (0.113426) | 2.40254 (0.066878) | 2.68043 (0.125097) | 0.0071 | <.0001 | 0.0029 | <.0001 | 0.0007 | 0.4542 |
| lh_entorhinal | 3.21977 (0.320881) | 3.17419 (0.344090) | 2.75954 (0.469706) | 3.34138 (0.350756) | 0.047 | 0.0072 | 0.9959 | 0.0037 | 0.4649 | 0.5987 |
| rh_entorhinal | 3.45989 (0.292824) | 3.31715 (0.365117) | 2.63454 (0.493994) | 3.59771 (0.315171) | 0.0017 | <.0001 | 0.3971 | 0.0001 | 0.046 | 0.449 |
| lh_bankssts | 2.39657 (0.129058) | 2.26112 (0.140998) | 2.14869 (0.190976) | 2.36048 (0.097197) | 0.2721 | 0.0018 | 0.0052 | 0.0123 | 0.084 | 0.7404 |
| rh_bankssts | 2.47937 (0.119088) | 2.33646 (0.164095) | 2.19177 (0.104657) | 2.42610 (0.168943) | 0.01 | <.0001 | 0.0017 | 0.001 | 0.1797 | 0.7929 |
| lh_parahippocampal | 2.47297 (0.253772) | 2.36342 (0.292375) | 2.21938 (0.315382) | 2.53005 (0.304518) | 0.3486 | 0.0275 | 0.423 | 0.0356 | 0.2353 | 0.915 |
| rh_parahippocampal | 2.52294 (0.182911) | 2.33954 (0.238076) | 2.21246 (0.251239) | 2.56705 (0.282282) | 0.5274 | 0.0054 | 0.0181 | 0.0123 | 0.0222 | 0.8526 |
|  | **Cortical thickness ^a^, mean (SD)** | | | | **p value ^b^** | | | | | |
|  | Cluster 1 (n=35) | Cluster 2 (n=26) | Cluster 3 (n=13) | Control (n=21) | C3 vs C2 | C3 vs C1 | C2 vs C1 | HC vs C3 | HC vs C2 | HC vs C1 |
| lh_insula | 2.93326 (0.123394) | 2.87065 (0.118767) | 2.73077 (0.171995) | 2.95486 (0.147531) | 0.1139 | 0.0036 | 0.2762 | 0.0051 | 0.172 | 0.918 |
| rh_insula | 2.94483 (0.141590) | 2.87246 (0.130112) | 2.71177 (0.211141) | 2.96948 (0.152188) | 0.1102 | 0.0044 | 0.1506 | 0.0123 | 0.1373 | 0.886 |
| lh_transversetemporal | 2.36631 (0.164905) | 2.18131 (0.164022) | 2.07815 (0.217270) | 2.24767 (0.136088) | 0.5274 | 0.0018 | 0.0011 | 0.187 | 0.3577 | 0.053 |
| rh_transversetemporal | 2.38000 (0.156394) | 2.22073 (0.219079) | 2.18946 (0.180704) | 2.41671 (0.166706) | 0.9952 | 0.0098 | 0.0159 | 0.0042 | 0.0127 | 0.8604 |
| lh_rostralmiddlefrontal | 2.32969 (0.082577) | 2.23850 (0.064596) | 2.14538 (0.079075) | 2.31995 (0.109450) | 0.0047 | <.0001 | 0.0004 | 0.0003 | 0.0269 | 0.9395 |
| rh_rostralmiddlefrontal | 2.34529 (0.086035) | 2.21031 (0.080523) | 2.13785 (0.065231) | 2.32700 (0.118913) | 0.0417 | <.0001 | <.0001 | 0.0002 | 0.0075 | 0.9344 |
| lh_caudalmiddlefrontal | 2.45780 (0.102908) | 2.35565 (0.112364) | 2.27177 (0.087355) | 2.42600 (0.116656) | 0.0993 | <.0001 | 0.0037 | 0.004 | 0.2084 | 0.7254 |
| rh_caudalmiddlefrontal | 2.48200 (0.108284) | 2.35062 (0.153316) | 2.28169 (0.093254) | 2.44719 (0.122407) | 0.2719 | <.0001 | 0.0073 | 0.0054 | 0.1135 | 0.8323 |
| lh_superiorfrontal | 2.69857 (0.097186) | 2.51850 (0.080913) | 2.45600 (0.120339) | 2.65252 (0.137472) | 0.1627 | <.0001 | <.0001 | 0.0025 | 0.0022 | 0.4234 |
| rh_superiorfrontal | 2.67663 (0.098963) | 2.53935 (0.093119) | 2.44815 (0.084955) | 2.65067 (0.132695) | 0.04 | <.0001 | <.0001 | 0.0006 | 0.0215 | 0.9292 |
| lh_parsopercularis | 2.50431 (0.114295) | 2.37569 (0.079531) | 2.32477 (0.139586) | 2.46648 (0.110992) | 0.688 | 0.0026 | <.0001 | 0.0413 | 0.0202 | 0.5176 |
| rh_parsopercularis | 2.50577 (0.112933) | 2.41650 (0.099819) | 2.31085 (0.133284) | 2.50714 (0.115778) | 0.0719 | 0.0006 | 0.0331 | 0.0015 | 0.0955 | 0.9993 |
| lh_parstriangularis | 2.43777 (0.121489) | 2.28746 (0.100054) | 2.22146 (0.117389) | 2.36905 (0.108894) | 0.3735 | 0.0001 | <.0001 | 0.0138 | 0.1136 | 0.2802 |
| rh_parstriangularis | 2.44534 (0.112990) | 2.31681 (0.078226) | 2.25723 (0.108870) | 2.38105 (0.099554) | 0.3166 | 0.0005 | 0.0003 | 0.0277 | 0.1505 | 0.2122 |
| lh_parsorbitalis | 2.68980 (0.154939) | 2.54646 (0.133236) | 2.44838 (0.236835) | 2.61314 (0.156346) | 0.2188 | 0.002 | 0.0058 | 0.0657 | 0.4517 | 0.4387 |
| rh_parsorbitalis | 2.73277 (0.165912) | 2.61838 (0.132240) | 2.44838 (0.187517) | 2.72348 (0.202539) | 0.0299 | 0.0001 | 0.0422 | 0.0048 | 0.2127 | 0.9846 |
| lh_lateralorbitofrontal | 2.58171 (0.110199) | 2.48742 (0.070192) | 2.37731 (0.123317) | 2.55414 (0.130975) | 0.0298 | 0.0002 | 0.0031 | 0.0025 | 0.2954 | 0.5177 |
| rh_lateralorbitofrontal | 2.51389 (0.108311) | 2.40081 (0.099721) | 2.37438 (0.138474) | 2.52133 (0.144102) | 0.7841 | 0.0087 | 0.0018 | 0.0339 | 0.0237 | 0.9958 |
|  |  |  |  |  |  |  |  |  |  |  |
|  | **Cortical thickness ^a^, mean (SD)** | | | | **p value ^b^** | | | | | |
|  | Cluster 1 (n=35) | Cluster 2 (n=26) | Cluster 3 (n=13) | Control (n=21) | C3 vs C2 | C3 vs C1 | C2 vs C1 | HC vs C3 | HC vs C2 | HC vs C1 |
| lh_medialorbitofrontal | 2.46694 (0.126865) | 2.35912 (0.117265) | 2.31915 (0.115546) | 2.41614 (0.181471) | 0.9027 | 0.0039 | 0.0047 | 0.6572 | 0.8327 | 0.2802 |
| rh_medialorbitofrontal | 2.41200 (0.138771) | 2.33938 (0.130653) | 2.24423 (0.102993) | 2.36486 (0.131171) | 0.1139 | 0.0029 | 0.2297 | 0.0688 | 0.9916 | 0.55 |
| lh_precentral | 2.45311 (0.118711) | 2.37150 (0.105271) | 2.27223 (0.135970) | 2.51295 (0.121879) | 0.1179 | 0.0014 | 0.0405 | 0.0007 | 0.0025 | 0.3231 |
| rh_precentral | 2.43514 (0.122521) | 2.34654 (0.095966) | 2.24254 (0.144969) | 2.47633 (0.094851) | 0.1102 | 0.0017 | 0.0631 | 0.0001 | 0.0003 | 0.615 |
| lh_paracentral | 2.28860 (0.119143) | 2.19127 (0.121558) | 2.10785 (0.182115) | 2.29486 (0.128590) | 0.2865 | 0.0059 | 0.0047 | 0.0192 | 0.0269 | 0.9983 |
| rh_paracentral | 2.34580 (0.133034) | 2.23796 (0.099681) | 2.19462 (0.148958) | 2.34595 (0.144736) | 0.8309 | 0.0127 | 0.0069 | 0.0477 | 0.0343 | 1 |
| lh_frontalpole | 2.78246 (0.211794) | 2.69650 (0.215384) | 2.63946 (0.241824) | 2.82514 (0.197252) | 0.954 | 0.4537 | 0.4405 | 0.0972 | 0.172 | 0.8365 |
| rh_frontalpole | 2.80114 (0.218905) | 2.67054 (0.163240) | 2.56162 (0.263103) | 2.78795 (0.170818) | 0.6788 | 0.0556 | 0.1167 | 0.0433 | 0.1506 | 0.9985 |
| lh_rostralanteriorcingulate | 2.67677 (0.187118) | 2.62877 (0.145244) | 2.63915 (0.284159) | 2.71786 (0.262751) | 0.9991 | 0.9031 | 0.6933 | 0.9072 | 0.6074 | 0.9987 |
| rh_rostralanteriorcingulate | 2.71620 (0.225131) | 2.58462 (0.155875) | 2.60338 (0.212023) | 2.67629 (0.183693) | 0.9968 | 0.327 | 0.0829 | 0.4445 | 0.3286 | 0.9466 |
| lh_caudalanteriorcingulate_ | 2.57389 (0.292156) | 2.48777 (0.219914) | 2.44423 (0.255240) | 2.54400 (0.213164) | 0.9921 | 0.5708 | 0.5922 | 0.8122 | 0.7645 | 0.9997 |
| rh_caudalanteriorcingulate | 2.44431 (0.222080) | 2.32773 (0.235970) | 2.16492 (0.312273) | 2.41705 (0.181766) | 0.1628 | 0.0084 | 0.3241 | 0.0213 | 0.655 | 0.9846 |
| lh_superiorparietal | 2.07403 (0.106052) | 1.96565 (0.072276) | 1.96938 (0.152414) | 2.04067 (0.114460) | 0.9702 | 0.1252 | <.0001 | 0.4445 | 0.2495 | 0.6525 |
| rh_superiorparietal | 2.07006 (0.096363) | 1.96585 (0.067848) | 1.95631 (0.137467) | 2.05476 (0.098103) | 0.9462 | 0.0325 | 0.0003 | 0.0931 | 0.0161 | 0.8364 |
| lh_inferiorparietal | 2.37789 (0.098129) | 2.22808 (0.077049) | 2.16154 (0.130771) | 2.37133 (0.122551) | 0.2315 | 0.0001 | <.0001 | 0.0011 | 0.0002 | 1 |
| rh_inferiorparietal | 2.36811 (0.083740) | 2.23554 (0.082385) | 2.15985 (0.130401) | 2.35286 (0.125319) | 0.2444 | 0.0001 | <.0001 | 0.0029 | 0.005 | 0.9856 |
| lh_supramarginal | 2.44103 (0.095917) | 2.32023 (0.083353) | 2.22223 (0.120545) | 2.40819 (0.138459) | 0.0594 | <.0001 | <.0001 | 0.0054 | 0.046 | 0.9208 |
| rh_supramarginal | 2.45040 (0.095308) | 2.30908 (0.090539) | 2.23077 (0.104309) | 2.42129 (0.123356) | 0.2007 | <.0001 | <.0001 | 0.0006 | 0.0043 | 0.5337 |
|  |  |  |  |  |  |  |  |  |  |  |
|  | Cluster 1 (n=35) | Cluster 2 (n=26) | Cluster 3 (n=13) | Control (n=21) | C3 vs C2 | C3 vs C1 | C2 vs C1 | HC vs C3 | HC vs C2 | HC vs C1 |
| lh_postcentral | 1.99529 (0.119518) | 1.88008 (0.081896) | 1.82338 (0.125042) | 1.96752 (0.111616) | 0.0774 | 0.0014 | 0.0005 | 0.0069 | 0.0435 | 0.7836 |
| rh_postcentral | 1.97566 (0.112035) | 1.84788 (0.066449) | 1.82392 (0.110630) | 1.91519 (0.086658) | 0.6414 | 0.0022 | <.0001 | 0.0413 | 0.0979 | 0.148 |
| lh_precuneus | 2.22740 (0.092547) | 2.09715 (0.072009) | 2.04008 (0.162244) | 2.22371 (0.122839) | 0.6881 | 0.0009 | <.0001 | 0.0098 | 0.0008 | 0.9993 |
| rh_precuneus | 2.25523 (0.085092) | 2.12758 (0.091619) | 1.99431 (0.140896) | 2.25043 (0.109044) | 0.0339 | <.0001 | <.0001 | 0.0005 | 0.0027 | 0.9876 |
| lh_posteriorcingulate | 2.42014 (0.114970) | 2.34812 (0.108198) | 2.26546 (0.130130) | 2.43200 (0.107355) | 0.0719 | 0.0035 | 0.0705 | 0.0012 | 0.128 | 1 |
| rh_posteriorcingulate | 2.35503 (0.123476) | 2.28477 (0.108147) | 2.15915 (0.098723) | 2.35057 (0.127750) | 0.004 | <.0001 | 0.2026 | 0.0042 | 0.2447 | 1 |
| lh_isthmuscingulate | 2.34737 (0.170191) | 2.24269 (0.144401) | 2.08815 (0.198986) | 2.30971 (0.140841) | 0.0719 | 0.0017 | 0.0668 | 0.0098 | 0.5118 | 0.7743 |
| rh_isthmuscingulate | 2.32637 (0.172207) | 2.20308 (0.144163) | 2.02800 (0.154493) | 2.19890 (0.174348) | 0.0201 | <.0001 | 0.0533 | 0.0393 | 0.9997 | 0.1507 |
| lh_lateraloccipital | 2.05586 (0.096681) | 1.91973 (0.089031) | 1.88023 (0.140765) | 2.04586 (0.161408) | 0.7331 | 0.0014 | <.0001 | 0.0433 | 0.0189 | 0.915 |
| rh_lateraloccipital | 2.10717 (0.089124) | 1.99015 (0.093014) | 1.94062 (0.120029) | 2.09690 (0.137234) | 0.4991 | 0.0006 | 0.0001 | 0.0172 | 0.0516 | 0.951 |
| lh_lingual | 1.87129 (0.088230) | 1.79954 (0.060213) | 1.68715 (0.119935) | 1.86505 (0.086740) | 0.01 | 0.0002 | 0.0091 | 0.0015 | 0.0123 | 1 |
| rh_lingual | 1.94634 (0.115466) | 1.85846 (0.082333) | 1.80946 (0.105017) | 1.90924 (0.085279) | 0.4803 | 0.0066 | 0.0135 | 0.0433 | 0.2352 | 0.8064 |
| lh_pericalcarine | 1.43660 (0.135894) | 1.39508 (0.096415) | 1.42762 (0.136628) | 1.44257 (0.109078) | 0.8081 | 1 | 0.7198 | 0.9771 | 0.3819 | 0.8406 |
| rh_pericalcarine | 1.46449 (0.140774) | 1.41623 (0.079060) | 1.41446 (0.049079) | 1.44795 (0.081911) | 0.9961 | 0.4118 | 0.5642 | 0.4335 | 0.5526 | 0.9917 |
| lh_cuneus | 1.72014 (0.083749) | 1.60438 (0.099119) | 1.73323 (0.202978) | 1.67462 (0.070295) | 0.195 | 1 | 0.0002 | 0.9742 | 0.0861 | 0.1858 |
| rh_cuneus | 1.78789 (0.117654) | 1.66485 (0.064155) | 1.71631 (0.111120) | 1.72629 (0.094990) | 0.3486 | 0.4187 | 0.0003 | 0.9996 | 0.1163 | 0.2761 |

Abbreviations: C1, Cluster 1; C2, Cluster 2; C3, Cluster 3; HC, Healthy control; SD, standard deviation.

^b^ P value was evaluated by Steel-Dwass test. Light gray indicates p value less than 0.0007.
